# Endophytic Cultivable Bacteria of the Metal Bioaccumulator *Spartina maritima* Improve Plant Growth but Not Metal Uptake in Polluted Marshes Soils

**DOI:** 10.3389/fmicb.2015.01450

**Published:** 2015-12-22

**Authors:** Jennifer Mesa, Enrique Mateos-Naranjo, Miguel A. Caviedes, Susana Redondo-Gómez, Eloisa Pajuelo, Ignacio D. Rodríguez-Llorente

**Affiliations:** ^1^Departamento de Microbiología y Parasitología, Facultad de Farmacia, Universidad de SevillaSevilla, Spain; ^2^Departamento de Biología Vegetal y Ecología, Facultad de Biología, Universidad de SevillaSevilla, Spain

**Keywords:** endophytes, heavy metal, phytoremediation, plant growth promoting bacteria (PGPB), salt marsh, *Spartina maritima*

## Abstract

Endophytic bacterial population was isolated from *Spartina maritima* tissues, a heavy metal bioaccumulator cordgrass growing in the estuaries of Tinto, Odiel, and Piedras River (south west Spain), one of the most polluted areas in the world. Strains were identified and ability to tolerate salt and heavy metals along with plant growth promoting and enzymatic properties were analyzed. A high proportion of these bacteria were resistant toward one or several heavy metals and metalloids including As, Cu, and Zn, the most abundant in plant tissues and soil. These strains also exhibited multiple enzymatic properties as amylase, cellulase, chitinase, protease and lipase, as well as plant growth promoting properties, including nitrogen fixation, phosphates solubilization, and production of indole-3-acetic acid (IAA), siderophores and 1-aminocyclopropane-1-carboxylate (ACC) deaminase. The best performing strains (*Micrococcus yunnanensis* SMJ12, *Vibrio sagamiensis* SMJ18, and *Salinicola peritrichatus* SMJ30) were selected and tested as a consortium by inoculating *S. maritima* wild plantlets in greenhouse conditions along with wild polluted soil. After 30 days, bacterial inoculation improved plant photosynthetic traits and favored intrinsic water use efficiency. However, far from stimulating plant metal uptake, endophytic inoculation lessened metal accumulation in above and belowground tissues. These results suggest that inoculation of *S. maritima* with indigenous metal-resistant endophytes could mean a useful approach in order to accelerate both adaption and growth of this indigenous cordgrass in polluted estuaries in restorative operations, but may not be suitable for rhizoaccumulation purposes.

## Introduction

Environmental pollution by heavy metals is a major concern for authorities (USEPA)^1^ due to several reasons: (i) its impact on environment and health, (ii) their high occurrence as a contaminant, (iii) their low solubility and bioavailability, and (iv) their carcinogenic and mutagenic nature (Davis et al., 2011). What is more, they cannot be degraded to harmless products and hence persist in the environment indefinitely (Khan and Doty, 2011). The environmental increase of heavy metal contamination is mainly due to industrial and agricultural activities (mining and smelting of metalliferous ores), waste water irrigation and chemical fertilizers and pesticides abuse (Bradl, 2002). The estuary of the Tinto and Odiel rivers, placed in the province of Huelva (Spain), is known as one of the most contaminated regions throughout the world due to the presence of high amounts of heavy metals in its sediments (especially As, Cu Pb, and Zn) since thousands of years (Nelson and Lamothe, 1993; Davis et al., 2000; Ruiz, 2001; Sáinz et al., 2002, 2004). This region has great environmental interest as well as historical significance (Wilson, 1981). The mining activity, mainly for Cu explotation, together with the later industrialization of the Huelva area, beginning in 1967, have contributed to the current level of pollution of this fluvial-estuarine system (Davis et al., 2000). By contrast, 30 km away from this estuary, is located the Piedras estuary, with absence of relevant anthropic contributions and no significant metallic pollution which therefore maintains its environmental quality (Borrego et al., 2013).

*Spartina maritima* (Curtis) Fernald belongs to the cordgrass family. It is an indigenous plant naturally growing in the estuary of three close rivers: Tinto, Odiel, and Piedras. It is distributed along the North-African and European coasts, playing an important role in the ecology of the saltmarshes, preserving the structure of intertidal coastal zones as well as defending the coast from erosion. What is more, a good tolerance of *S. maritima* to anthropogenic pollutants, tidal submergence, salinity and drainage has been demonstrated (Mateos-Naranjo et al., 2007, 2010). The utility of this species for biomonitoring coastal systems where it is abundant has also been described (Padinha et al., 2000). On top of that, heavy metals accumulation at different rates in *S. maritima* tissues and rhizosediment allowed concluding that this species could be used for phytostabilization of estuarine sediments (Cambrollé et al., 2008; Redondo-Gómez, 2012). Unfortunately, the invasive *Spartina densiflora* is displacing the native *S. maritima* and colonizing non-restored marshes of the southwest coast of Spain (Castillo et al., 2008). In that situation, it is important not only to learn how to efficiently rehabilitate degraded salt marshes but also how to adequately manage populations of *S. densiflora*, preserving the endangered native plant population (An et al., 2007; Mateos-Naranjo et al., 2012).

In soil metal phytoremediation, plants should be capable to handle with large amounts of heavy metals and reach a high biomass at the same time. Nevertheless, their growth may be limited at high pollution rates, resulting in small size and slow growth rate, lessening their phytoextraction capacity (reviewed in Ali et al., 2013). So that, the beneficial relationships between plants and their associated microbes could be exploited to expedite the production of plant biomass and thereby determine plant metal stabilization (Glick, 2010; Ma et al., 2011; Rajkumar et al., 2012). There is an increasing literature reporting the effect of bacterial inoculation on plant growth and metal uptake. In all these studies, plant-associated bacteria increased plant growth but the effect on metal uptake depended on the specific plant-microorganism partnerships and also on the soil characteristics (Sessitsch et al., 2013; Phieler et al., 2014). Metal bioavailability is often plant species and element specific, and is clearly influenced by metabolites excreted by applied bacteria (Tak et al., 2013; Langella et al., 2014). It has been recently suggested that interactions between halophyte and microorganisms could be useful in phytoremediation strategies in coastal ecosystems (Reboreda and Caçador, 2008; Andrades-Moreno et al., 2014; Mesa et al., 2015b). Although interactions between plants and microbes in the rhizosphere have long been studied by microbial ecologists (de-Bashan et al., 2012), plant growth promoting bacterial (PGPB) endophytes may offer several advantages and are gaining scientific interest (reviewed in Rajkumar et al., 2009). Concerning the genus *Spartina*, several works about their rhizospheric bacterial populations have been published (Lovell et al., 2000; Gamble et al., 2010; Davis et al., 2011; Andrades-Moreno et al., 2014; Mesa et al., 2015a,b), but endophytic bacteria have never been characterized so far.

The aims of this work were (i) to isolate the cultivable endophytes from *S. maritima* growing in salt marshes with different levels of metal contamination, (ii) to characterize them and select the endophytic PGPBs which might be useful for increasing plant biomass production, and (iii) to inoculate wild *S. maritima* seedlings in greenhouse conditions to elucidate the influence of these PGPBs in plant metal uptake in contaminated soils.

## Materials and methods

### Plant and soil sampling and chemical analysis

Plant samples of *S. maritima* were harvested in May 2013 from the Tinto (37°13′ N, 6°53′ W), Odiel (37°10′35.2″N 6°55′59.2″W) and Piedras (37°16′09.1″N 7°09′36.4″W) rivers estuaries. Soil samples were collected in the same locations. They were transported to the laboratory and stored at 4°C. Endophytes were isolated within 24 h. For chemical analysis, plant tissues were carefully washed with distilled water and dried at 80°C for 48 h, grounded and homogenized (Redondo-Gómez et al., 2007). Then, samples were acid-digested and the cool residue extracted as described in Cambrollé et al. (2008). Inductively coupled plasma (ICP-AES) spectroscopy (ARLFisons3410, USA) was used to measure elements concentration. Concentrations were expressed as mg/Kg (Table 1).

**Table 1:** **Metal(loid) concentrations in mg/Kg for soil samples and *S. maritima* tissues in Tinto, Odiel and Piedras rivers estuaries**.

	**Location**	**As**	**Cd**	**Co**	**Cu**	**Fe**	**Mn**	**Ni**	**Pb**	**Zn**
Root	Tinto	160.54 ± 1.44^a^	2.15 ± 0.02^a^	11.57 ± 0.10^a^	545.47 ± 4.88^a^	21797.15 ± 194.96^a^	53.40 ± 0.48^a^	5.74 ± 0.05^a^	60.00 ± 0.54^a^	428.73 ± 3.83^a^
	Odiel	98.94 ± 0.88^b^	2.06 ± 0.02^b^	12.38 ± 0.11^b^	251.41 ± 2.25^b^	18850.33 ± 168.60^b^	124.40 ± 1.11^b^	6.74 ± 0.06^b^	72.78 ± 0.65^b^	779.93 ± 6.98^b^
	Piedras	29.71 ± 0.27^c^	0.54 ± 0.00^c^	2.88 ± 0.03^c^	62.37 ± 0.56^c^	7162.19 ± 64.06^c^	42.01 ± 0.38^c^	7.04 ± 0.06^c^	20.80 ± 0.19^c^	180.91 ± 1.62^c^
Stem	Tinto	4.95 ± 0.04^a^	0.24 ± 0.00^a^	< 0.5 ± 0.00^a^	74.27 ± 0.66^a^	672.77 ± 6.02^a^	34.20 ± 0.31^a^	1.29 ± 0.01^a^	6.87 ± 0.06^a^	59.44 ± 0.53^a^
	Odiel	2.91 ± 0.03^b^	0.38 ± 0.00^b^	< 0.5 ± 0.00^a^	39.47 ± 0.35^b^	1047.61 ± 9.37^b^	73.85 ± 0.66^b^	1.68 ± 0.01^b^	9.53 ± 0.09^b^	96.24 ± 0.86^b^
	Piedras	0.47 ± 0.00^c^	< 0.1 ± 0.00^c^	< 0.5 ± 0.00^a^	3.74 ± 0.03^c^	602.89 ± 5.39^c^	46.88 ± 0.42^c^	1.69 ± 0.02^b^	1.14 ± 0.01^c^	26.02 ± 0.23^c^
Leaf	Tinto	19.68 ± 0.18^a^	0.19 ± 0.00^a^	< 0.5 ± 0.00^a^	98.01 ± 0.88^a^	2692.89 ± 24.09^a^	53.58 ± 0.48^a^	2.40 ± 0.02^a^	25.68 ± 0.23^a^	105.26 ± 0.94^a^
	Odiel	9.09 ± 0.08^b^	0.17 ± 0.00^b^	0.54 ± 0.00^b^	51.35 ± 0.46^b^	3303.44 ± 29.55^b^	141.32 ± 1.26^b^	3.49 ± 0.03^b^	23.80 ± 0.21^b^	123.98 ± 1.11^b^
	Piedras	1.04 ± 0.01^c^	< 0.1 ± 0.00^c^	< 0.5 ± 0.00^a^	7.46 ± 0.07^c^	1431.08 ± 12.80^c^	62.53 ± 0.56^c^	3.08 ± 0.03^c^	3.46 ± 0.03^c^	28.03 ± 0.25^c^
Soil	Tinto	546.0 ± 4.89^a^	5.23 ± 0.05^a^	20.54 ± 0.18^a^	2460.01 ± 22.01^a^	106231.78 ± 950.17^a^	326.53 ± 2.92^a^	43.49 ± 0.39^a^	701.35 ± 6.27^a^	2523.69 ± 22.57^a^
	Odiel	127.0 ± 1.14^b^	3.49 ± 0.03^b^	5.24 ± 0.05^b^	889.11 ± 7.93^b^	69117.33 ± 537.71^b^	472.20 ± 4.22^b^	29.73 ± 0.27^b^	275.81 ± 2.47^b^	1655.23 ± 14.80^b^
	Piedras	3.1 ± 0.03^c^	0.18 ± 0.00^c^	< 0.5 ± 0.00^c^	20.99 ± 0.19^c^	12707.28 ± 113.66^c^	75.02 ± 0.67^c^	10.65 ± 0.10^c^	21.12 ± 0.19^c^	71.63 ± 0.264^c^

Values are means ± s.e. (n = 5). Different letters indicate statistical differences for each metal(loid) and tissue between locations (P < 0.05).

### Isolation and morphologic characterization of cultivable endophytic bacteria from *S. maritima* tissues

Endophytic bacteria were isolated from surface-sterilized leaves, stems and roots of *S. maritima*. The protocol of surface-sterilized was as follows: first surface washing under running tap water to remove soil, insects and other big particles, followed by immersion in 70% (v/v) ethanol for 2 min under slightly shaking, then soaking in 5% (v/v) sodium hypochlorite for 10 min under gentle agitation, and finally 5 rinses in sterile distilled water. Several controls confirmed that the sterilization procedure was effective. On one hand, fragments from each tissue were transferred to commercial tryptic soy agar (TSA) medium (iNtRON Biotechnology, Korea) and modified TSA medium NaCl 0.6 M (approximately seawater salt concentration). On the other hand, duplicates of 100 μL aliquots of the last washing water were also plated in the same way. No bacteria were grown within 5 days incubation at 28°C.

Leafs and roots were macerated separately using a sterile mortar and pestle in a small volume of sterile physiological saline solution, with sterile quartz sand being added to improve the wall disruption. The stems were placed inside sterile pipette tips and then into sterile Falcon tubes. They were centrifuged at 1500 rpm for 5 min, and the obtained liquid was discarded. Then, they were spun at 5000 rpm during 20 min, and the apoplastic fluid was collected. 100 μL of the three resulting tissue extracts were plated onto TSA and TSA 0.6 M NaCl plates, in order to recover halophilic bacteria. Following the incubation during 72–96 h at 28°C, colonies of varying morphology were picked and then subsequently re-streaked on TSA and modified TSA NaCl 0.6 M in order to obtain pure cultures. The morphological characterization was carried out by recording colony characters based on shape, margin, color, surface and consistency followed by Gram staining. TSA NaCl 0.6 M was prepared as described in Mesa et al. (2015a).

### Genetic diversity by BOX-PCR

Genomic DNA extraction and BOX-PCR were performed exactly as described in Mesa et al. (2015a). For BOX-PCR, the BOX A1R primer (5′- CTA CGG CAA GGC GAC GCT GAC G -3′) and 40 ng of DNA as template were used. After PCR, products were electrophoresed using an agarose gel (1.5%) and revealed by UV radiation. The gel was photographed and the images were processed with Phoretix 1D® Software (TotalLab, UK), resulting in dendograms. The similarities in BOX-PCR fingerprints were established by determining the Pearson's product moment correlation coefficient (Jobson, 1991).

### Identification of cultivable bacteria

The bacteria with most interesting properties were selected for PCR amplification of conserved 16S rRNA and subsequent sequencing and analysis. For PCR amplification, universal primers 16S F8-27 (5′-AGAGTTTGATCCTGGCTCAG-3′) and 16S R1541-1522 (5′-AAGGGAGGTGATCCAGCCGCA-3′) were employed. The reaction mixture contained for 25 μl: 10x Ecogen buffer 2.5 μl, MgCl_2_ 50 mM 1 μl, dNTPs 10 mM (2.5 mM each) 1 μl, EcoTaq polymerase (5U/μl) 0.2 μl, extracted DNA 1 μl, each primer 10 μM 1 μl and H_2_O_MQ_17.3 μl. The temperature profile was programmed as follows: predenaturation at 95°C for 2 min, 35 cycles of denaturation at 95°C for 45 s, annealing at 58°C for 45 s, extension at 72°C for 90 s (35 cycles) and final extension at 72°C for 5 min. The amplified products were checked by running on 1% agarose gel and visualized under UV after staining with RedSafe™ Nucleic Acid Staining Solution (iNtRON Biotechnology, Korea). The PCR product was purified with SpeedTools PCR Clean-up kit (Biotools, Spain) and sequencing was done by StabVida Company (Portugal). The EzTaxon server was used to determine 16S rRNA sequence homologies (Chun et al., 2007). Finally, accession numbers from KT036396 to KT036409 were assigned to the sequences deposited in GenBank (Table 2).

**Table 2:** **Closest species to the fourteen isolates based on the 16S rRNA sequence**.

**Strain**	**16S rRNA sequenced fragment (bp)**	**Accession No**.	**Related species**	**Percent identity**
SMJ1	1413	KT036396	*Staphylococcus warneri*	99.64
SMJ4	1424	KT036397	*Micrococcus yunnanensis*	99.58
SMJ12	1430	KT036398	*Micrococcus yunnanensis*	99.58
SMJ15	1483	KT036399	*Bacillus selenatarsenatis*	98.09
SMJ17	1458	KT036400	*Bacillus aryabhattai*	99.59
SMJ18	1405	KT036401	*Vibrio sagamiensis*	98.46
SMJ19	1418	KT036402	*Marinomonas alcarazii*	96.18
SMJ20	1352	KT036403	*Marinomonas ostreistagni*	97.40
SMJ24	1407	KT036404	*Vibrio sagamiensis*	98.76
SMJ25	1454	KT036405	*Pseudoalteromonas shioyasakiensis*	99.72
SMJ28	1453	KT036406	*Marinomonas alcarazii*	96.18
SMJ30	1440	KT036407	*Salinicola peritrichatus*	97.29
SMJ32	1347	KT036408	*Salinicola peritrichatus*	97.84
SMJ33	1462	KT036409	*Staphylococcus pasteuri*	99.51

### Bacterial resistance against NaCl and metal(loid)s

The resistance of isolated bacteria toward heavy metals and sodium arsenite was determined on plates containing TSA and modified TSA 0.2M NaCl mediums amended with the following heavy metal stock solutions were employed: CuSO_4_ 1 M, ZnSO_4_ 1 M, NiCl_2_ 0.2 M, CoCl_2_ 1 M, CdCl_2_1 M, Pb(NO_3_)_2_0.5 M, and NaAsO_2_ 0.5 M, as detailed in Mesa et al. (2015a). To establish NaCl tolerance, SW30 stock solution was added to TSA medium. The resistance was expressed as the maximum tolerable concentration (MTC), namely the highest metal or metalloid concentration not impeding bacterial growth.

### Screening for bacterial enzyme activity

Strains were tested for amylase, cellulase, lipase, protease, and chitinase activities. They were screened in plates. To detect amylase activity, the isolates were inoculated on starch agar (Scharlab, Spain), and were revealed after incubation by flooding the plates with iodine–potassium iodide solution (lugol). In the case of cellulase, strains were plated onto solid minimal medium M9 supplemented with 0.2% yeast extract and 1% carboxymethyl cellulose (CMC) and was revealed after incubation by flooding the plates with Congo Red solution 1mg/ml for 15 min followed by destaining with sodium chloride 1 M for 15 min. Protease activity was detected by growing the strains in casein agar (Prescott, 2002). All the plates were incubated for 5 days at 28°C and observed for clear zones around the cultures. In the case of lipase activity, strains were grown in Tween agar (Prescott, 2002), plates were incubated for 7 days at 28°C, and looked for the appearance of a precipitate around the strains. Chitinase activity was tested using a minimal medium (per liter, 2.7 g K_2_HPO_4_, 0.3 g KH_2_PO_4_, 0.7 g MgSO_4_ · 7H_2_O, 0.5 g KCl, 0.13 g yeast extract, 15 g agar, H_2_O e.q. to 1 L, pH 7.2) supplemented with colloidal chitin (1.5%). The plates were incubated at 28°C for 7 days until zones of chitin clearing could be seen around the colonies. NaCl concentration in all media was adjusted to 0.2 M by adding filter sterilized SW30 solution after autoclaving to avoid Ca precipitation.

### Screening for bacterial plant growth promoting traits

Bacterial plant growth promoting traits were recorded as described in Mesa et al. (2015a). Bacterial growth in NFb medium was used to test nitrogen fixation (Dobereiner, 1995). Phosphate solubilization was confirmed on NBRIP medium plates (Nautiyal, 1999) when bacterial growth caused the appearance of surrounding transparent halos. In the same way, orange halos revealed production of siderophores on chrome azurol S (CAS) plates (Schwyn and Neilands, 1987). Plates were always incubated 72 h at 28°C. The synthesis of IAA (indole-3-acetic acid) was colorimetrically estimated as detailed in Mesa et al. (2015a). Presence of ACC deaminase enzyme was detected following the method described in Penrose and Glick (2003). Liquid Dworkin and Foster (DF) mineral medium (Dworkin and Foster, 1958) with 3.0 mM ACC was used to inoculate endophytic strains after enrichment with the same medium with (NH_4_)_2_SO_4_ as nitrogen source instead of ACC. The growth on the tubes was checked daily during 3 days at 28°C. As mentioned before, NaCl concentration in all media was adjusted to 0.2 M by adding filter sterilized SW30 solution after autoclaving. Based on the results from this experiment, ACC deaminase activity was determined by monitoring the amount of α-ketobutyric acid generated from the cleavage of ACC (Penrose and Glick, 2003). The reaction was determined by comparing the absorbance at 540 nm of the sample to a standard curve of α-ketobutyrate. Then, total protein concentration of toluenized cells (Bradford, 1976) was estimated using bovine serum albumin (BSA) to produce the protein calibration curve. After determining the amount of protein and α-ketobutyrate, the enzyme activity was calculated based on the μmoles of released α-ketobutyrate per mg of protein per hour.

### Pot inoculation in greenhouse conditions

In April 2014, wild plants along with wild soil were collected from the Tinto River estuary, the most contaminated. They were carried in pots, filled with 1 Kg of soil from the marsh, to the greenhouse facilities at the University of Seville. Pots were randomly assigned to two treatments (non-inoculated control plants; inoculated plants) and placed in the same greenhouse for 30 days (*n* = 20, two treatments with 10 pots each one). During the experimental period the inoculations were performed once a week (four times in total). For that, bacteria were grown separately in 250 ml Erlenmeyer flasks containing 50 ml of TSB medium (iNtRON Biotechnology, Korea) with continuous gentle shaking at 28°C to reach 10^8^ cells per ml (18–24 h). Then, cultures were centrifuged at 8000 rpm during 10 min, the supernatant was discarded and pellets were resuspended in 2 L tap water. Hundred milli liter of suspension per pot were used for plant inoculation. Pots were placed in trays (different trays for each treatment) and were lightly watered with tap water every 2 days during the experiment to avoid dryness, since *S. maritima* is a low marsh plant used to tidal flooding.

### *S. maritima* re-colonization potential of the endophytic consortium

In parallel to inoculation treatments, three plants were inoculated with mutant endophytic PGPB in order to confirm they penetrate and colonize plant tissues. *Salinicola* SMJ30 was labeled with the fluorescent protein mCherry by bacterial conjugation. Strains and plasmids used are listed in Table 3. This was accomplished by mixing the donor *E. coli* DH5α containing pMP7604, the helper *E. coli* strain containing pRK600 and the recipient strain *Salinicola* SMJ30 in liquid LB medium overnight under slightly shaking at 28°C. Next, 100 μL aliquots were plated onto LB plates containing selective antibiotic for *Salinicola* SMJ30 (rifampicin) and for pMP7604 plasmid (tetracycline). For wild endophytes *Micrococcus* SMJ12 and *Vibrio* SMJ18 no transformants were detected with conjugation, electroporation, heat-shock or freeze-thaw techniques, even with lysozyme pre-treatments. Thus, spontaneous rifampicin and streptomycin resistant mutants of SMJ12 and SMJ18 wild strains were used. They were developed by transfer of high concentrated bacterial overnight liquid cultures in TSA plates amended with 100 μg ml^−1^ of rifampicin and 250 μg ml^−1^ of streptomycin. The mutant strains showing comparable growth with wild type strains were selected. After the inoculation experiment, fluorescent bacteria in plant tissues were visualized in a confocal microscope (Zeiss LSM 7 DUO). Images were obtained using laser excitation 568–585 nm long pass emission and were processed with ZEN Lite 2012 software. Furthermore, 20 days after the last inoculation, plant tissues and soil samples were collected. 100 mg of plant tissues were surface-sterilized and macerated as explained in 2.2. Regarding soil, 100 mg were resuspended in 500 μl sterile physiological saline solution. 100 μl of the obtained extracts were plated into rifampicin and tetracycline plates (for detection of strain SMJ30), rifampicin and streptomycin plates (for strains SMJ12 and SMJ18) and incubated 72 h at 28°C. Colonies obtained were identified by macroscopic and microscopic observation (colony shape, surface and color, Gram staining and determination of motility in a wet mount) and API® identification products (bioMérieux, France) (API® 20 NE for SMJ30 and SMJ18, API® Staph for SMJ12). Then, colonies were counted to estimate colony-forming units (CFU) per gr of tissue or soil.

**Table 3:** **Strains and plasmids used for bacterial conjugation**.

**Strain or plasmid**	**Relevant characteristics**	**Reference or source**
***Escherichia coli***		
DH5α	Host strain used for transformation and propagation of plasmids containing pMP7604, Tc^r^	Boyer and Roulland-Dussoix, 1969
DH5α	Helper strain used for propagation of plasmids containing pRK600, Cm^r^	Kessler et al., 1992
***Salinicola peritrichatus***		
SMJ30	Endophyte isolated from *Spartina maritima* stems, Rif^r^	This study
**PLASMIDS**		
pMP7604	pMP6031 derivate harboring mCherry gene under the control of the tac promoter	Lagendijk et al,.2010
pRK600	Cm^r^ Nm^s^, pRK2013 Nmr::Tn9	Finan et al., 1986

### Plant growth and physiological analysis

At the beginning and at the end of the experiment, three and ten plants per treatment were harvested and dried at 80°C to estimate roots and shoots dry weights and calculate the relative growth rate, RGR (Mateos-Naranjo et al., 2008). Also 30 days after treatment initiation leaf gas exchange and chlorophyll fluorescence parameters were measured in fully expanded leaves (*n* = 10) using an infrared gas analyzer (LI-6400-XT, Li-COR Inc., NE., USA) and a modulate fluorimeter (FMS-2; Hansatech Instruments Ltd., UK), respectively. Thus, net photosynthetic rate (A_N_), stomatal conductance (gs), instantaneous water use efficiency (iWUE) and intercellular CO_2_ concentration (C_i_) were obtained with the following settings: flux light density1500 μmol photons m^−2^ s^−1^, ambient CO_2_ concentration (Ca) 400 μmol mol^−1^ air, leaf temperature of 25°C and 50 ± 5% relative humidity. Minimal fluorescence (F_0_), maximum quantum efficiency of PSII photochemistry (F_v_/F_m_) and quantum efficiency of PSII (Φ_PSII_) were obtained in light and dark-adapted leaves at midday (1600 μmol photons m^−2^ s^−1^) according to the protocol described by Mateos-Naranjo et al. (2008).

### Chemical analyses of plant tissues

At the end of the experiment, 30 days after treatment initiation, dried leaves and root samples of the 10 replicates plants were ground, and total As, Cd, Cu, Ni, Pb, and Zn concentrations were measured as previously described by Mateos-Naranjo et al. (2008) by inductively coupled plasma (ICP) spectroscopy.

Metal balance in plant and soil was calculated as the ratio between metal accumulated in plant tissues (metal concentration in shoots × shoot biomass + metal concentration in roots × root biomass) with regard to metal in soil. This ratio was estimated both at the beginning and at the end of the experiment.

### Statistical analysis

Statistical analyses were carried out using “Statistica” v. 6.0. Comparisons between means of metal(loid) concentration for *S. maritima* tissues in Tinto, Odiel and Piedras rivers estuaries were made by using one-way anova (*F*-test) and between means in different inoculation treatments at the end of the experiment through the Student test (*t*-test).

## Results

### Concentration of metal(loid)s in *S. maritima* tissues in the tinto, odiel, and piedras rivers estuaries

Concentration of metals and metalloids in different tissues of *S. maritima* in the three estuaries were determined (Table 1). They were similar to data previously published by other authors (reviewed in Redondo-Gómez, 2012). Depending on their final use, there are threshold values for metals in soils (but not for plant tissues) established by Andalusian and Spanish Governments (Consejería de Medio Ambiente, Junta de Andalucía, 1999). Generally, the Tinto salt marsh showed the higher metal concentration, except for Mn, Ni and Zn, greater in Odiel estuary. Regarding plant tissues, metal levels were higher in roots in all the estuaries.

### Isolation of cultivable endophytic bacteria from *S. maritima* tissues in the tinto, odiel, and piedras rivers salt marshes

**Figure 1:**
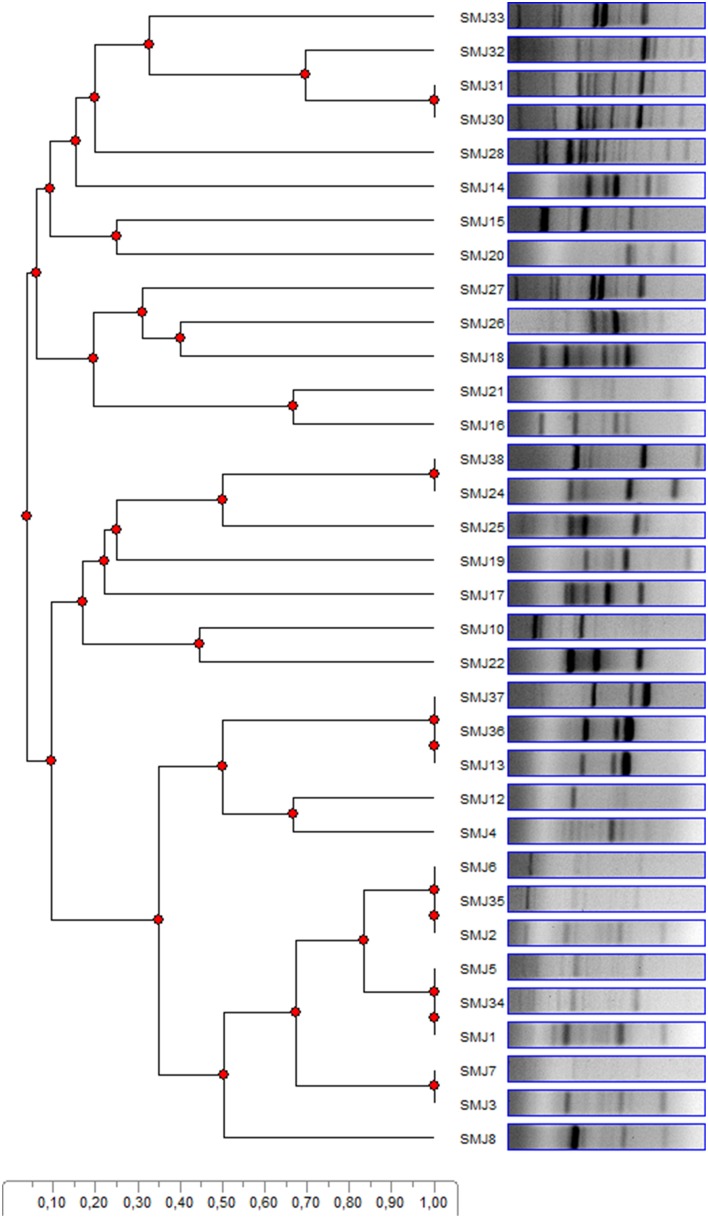
**BOX-PCR patterns of the 34 endophytic strains from *S. maritima* tissues in the Tinto, Odiel and Piedras rivers estuaries are compared using a dendogram**. Similarity is represented by the scale.

Bacteria grown on TSA plates showing different colony morphologies were chosen. This resulted in 42 strains. Unfortunately, several strains could not be systematically re-isolated in standard media and could not be characterized properly (Hardoim et al., 2008). Several strains showed identical basic morphology properties; therefore, the number of different strains was reduced to 34. Subsequently, a BOX-PCR was performed in order to avoid redundancy. As shown in Figure 1, few bacteria showed identical band profile, and were then discarded. Despite the fact that some strains showed similar profiles, further analyses during the development of this work (see results below) demonstrated distinctive differences. Thus, they were considered different strains. As a result, the scope of the study was finally reduced to 25 strains. For bacterial identification of the strains with most interesting properties, 16S rRNA genes were partially sequenced (Table 2).

### Distribution of cultivable endophytic bacteria

**Figure 2:**
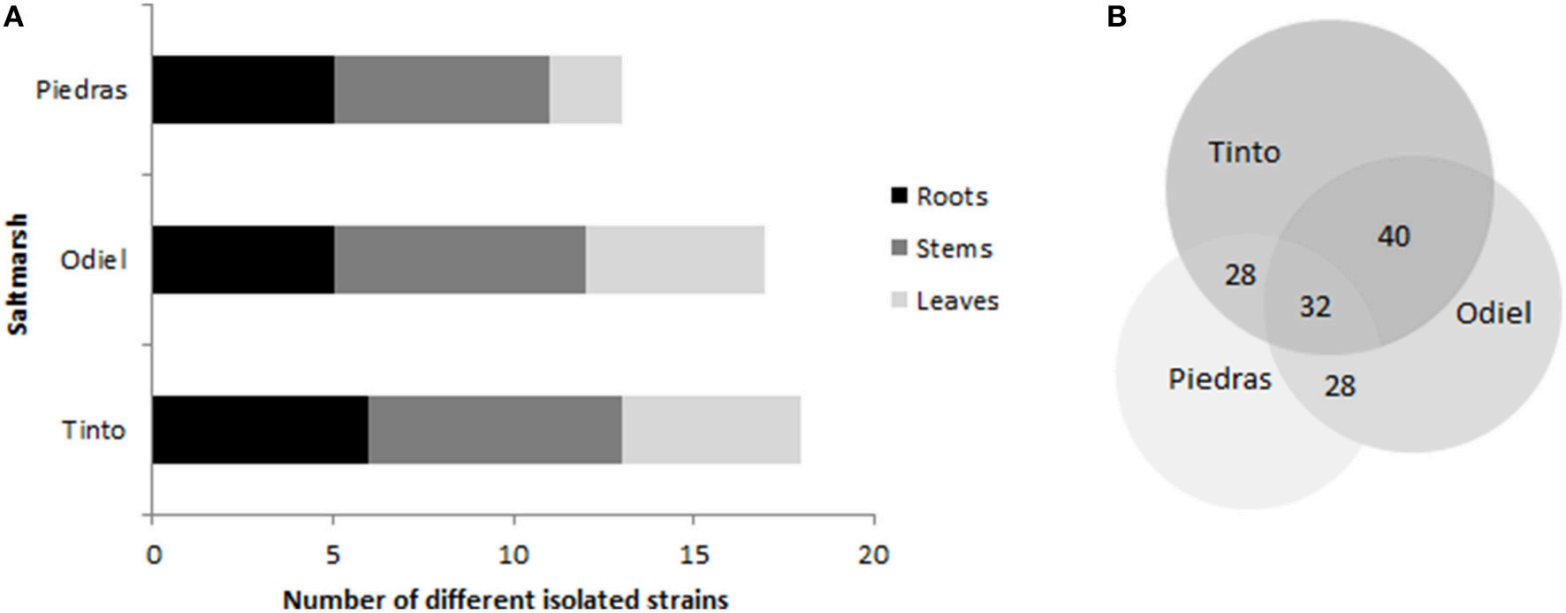
**Chart illustrating (A) cultivable endophytes location within distinct plant tissues in each estuary and (B) cultivable endophytes distribution according to the salt marshes expressed as a percentage of total strains isolated (25)**.

Distribution of endophytic strains is represented in Figure 2. *S. maritima* from Piedras estuary, the less contaminated saltmarsh, showed the scantiest cultivable endophytic diversity (13 strains), while the ones located in the most contaminated area, the Tinto estuary, presented the largest heterogeneity (18 strains), similarly to Odiel estuary (17 strains). Regarding plant tissues (Figure 2A), stems harbored from 39 to 46% of the cultivable strains, followed by roots with the 30 and 38%. In contrast, leaves had only 16–29%. Finally, concerning the distribution of cultivable endophytes between different estuaries, 8 out of 25 strains (32%) were common in the three estuaries with different level of pollution (Figure 2B). When comparing the estuaries, Tinto and Odiel shared 40% of cultivable endophytes, being the most similar. The origin of each isolate (estuary and plant tissue) is illustrated in Supplementary Table 1.

### Endophytes abiotic stress resistance toward NaCl and heavy metals

**Figure 3:**
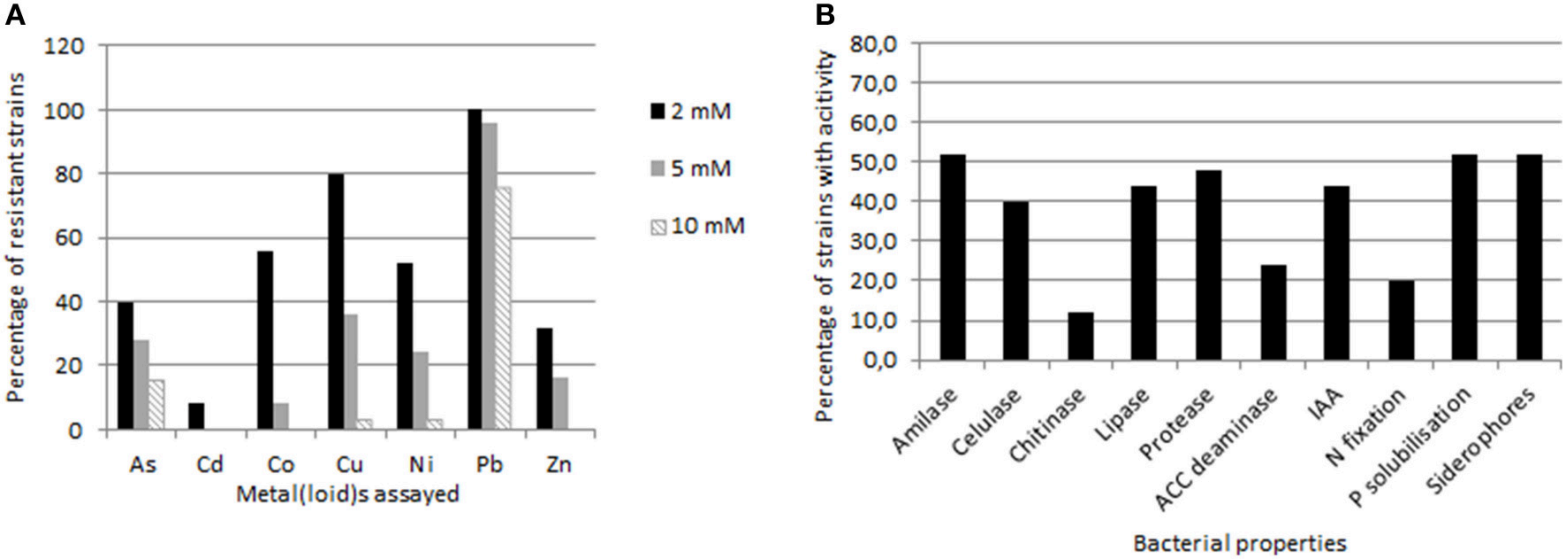
**Graphics showing percentages of studied strains (A) with resistance to different heavy metals over concentrations of 2 mM, 5 mM or 10 mM, and (B) with enzymatic and plant growth promoting properties**.

Resistance to NaCl as well as to several heavy metals (As in sodium meta-arsenite form, Cd, Co, Cu, Ni, Pb, and Zn) was established and represented as maximum tolerable concentration (MTC, the maximum concentration that allows bacterial visible growth) for each strain (Supplementary Table 2). Data are summarized in Figure 3A. Resistance to various metals simultaneously was observed for the majority of the isolates, including noteworthy resistance values in several cases. Pb was the most tolerated metal; all the strains resisted over 2 mM, getting to 25 mM for strains SMJ1, SMJ2, SMJ3, SMJ30, SMJ32, and SMJ33. This was followed by Cu, 80% of the strains resisted over 2 mM Cu_2_SO_4._Strains SMJ4, SMJ12, and SMJ33 were resistant up to 8–9 mM Cu. Around 50% of the strains were resistant toward Co and Ni as well. Concerning As, some strains presented a high resistance, SMJ10 and SMJ17 arrived to 13 mM, SMJ3 reached 25 mM and the most striking result was SMJ12 reporting resistance to 100 mM NaAsO_2_. By contrast, the resistance toward Cd of the isolates was not high (scarcely 2 mM). Finally, NaCl tolerance was also studied (supplementary material), which ranged from 0.5 to 3 M, hence most of isolated bacteria could be considered halotolerant.

### Enzymatic and PGP properties of the endophytic isolates

The percentage of endophytic strains showing the different enzymatic and PGP properties studied is presented in Figure 3B. Regarding enzymatic activities, 21 out of 25 strains exhibited at least one enzyme activity (Supplementary Material). Amilase production was the most common between the isolates (52%), followed by protease (48%), lipase (44%), cellulase (40%), and finally chitinase activity (12%). Notably, strains SMJ4, SMJ14, SMJ17, SMJ20, SMJ21, and SMJ24 had at least 3 of the 5 enzymatic properties assayed, whereas strains SMJ18 and SMJ25 showed all of them. On top of that, SMJ18 produced the biggest halos in plates assay for all the properties. Moving to PGP properties, represented in the same figure as data above, 76% of the strains had at least one PGP property. Overall, 52% were able to solubilize phosphate and produce siderophores, 44% produced IAA, 20% fixed atmospheric nitrogen and only 8% hydrolysed ACC. Concerning this last one, it is important to measure the enzymatic activity, since some strains were able to grow in the minimal medium with ACC and later gave false positives results. Seven strains were able to grow in the presence of ACC, but only 2 revealed enzymatic activity in the colorimetric assay. Only SMJ28 showed all the properties assayed, while 19% of the strains (SMJ12, SMJ17, SMJ30, and SMJ32) presented 4 out of five properties. SMJ12, SMJ18, and SMJ20 were found to be prominent IAA producers (4.83, 4.52, and 5.18 mg/ml respectively) whereas SMJ30 managed the best siderophores formation capacity and SMJ32 the most notably phosphate solubilization.

### Selection of the best-performing endophytic strains for pot inoculation under greenhouse conditions

*Micrococcus yunnanensis* SMJ12, *Vibrio sagamiensis* SMJ18, and *Salinicola peritrichatus* SMJ30 were selected as the best-performing strains, based on their enzymatic and PGP properties, heavy metals resistance and salt tolerance. While SMJ12 managed the best auxins production and had a marked resistance to As and Cu, SMJ30 had a powerful siderophores formation capacity as well as P solubilization, demonstrating high tolerance to Zn, Pb, and NaCl. Finally, strain SMJ18 was selected by its auxins production and its prominent enzymatic properties, together with a notable resistance to Ni and Co. The three bacterial isolates were cultivated together and no antagonistic activity between them was observed (data not shown). Despite that SMJ28 presented all the PGP properties studied, including the beneficial ACC deaminase activity, its general metal resistance was very poor, probably because it was isolated from the non-contaminated estuary. Then, it was not considered for this bacterial consortium.

### Effect of inoculation on plant growth and physiological parameters

**Figure 4:**
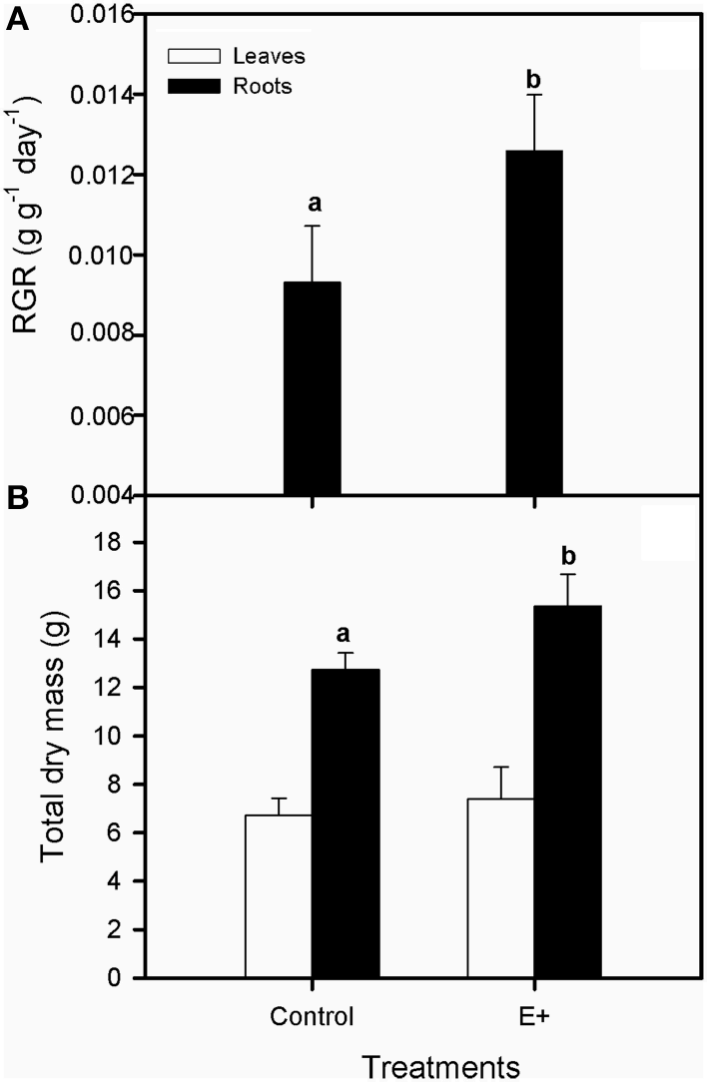
**Effect of inoculation (control, without inoculation; E+, inoculations repeated once a week during experimental period) with a bacterial consortium integrated by *Micrococcus yunnanensis* SMJ12, *Vibrio sagamiensis* SMJ18 and *Salinicola peritrichatus* SMJ30 on relative growth rate, RGR (A) and aboveground biomass and belowground biomass (B) in *Spartina maritima* plants grown in natural soil from Tinto marsh for 30 days**. Values are means ± s.e. (*n* = 10). Statistical differences between means are indicated by different letters (*P* < 0.05).

**Figure 5:**
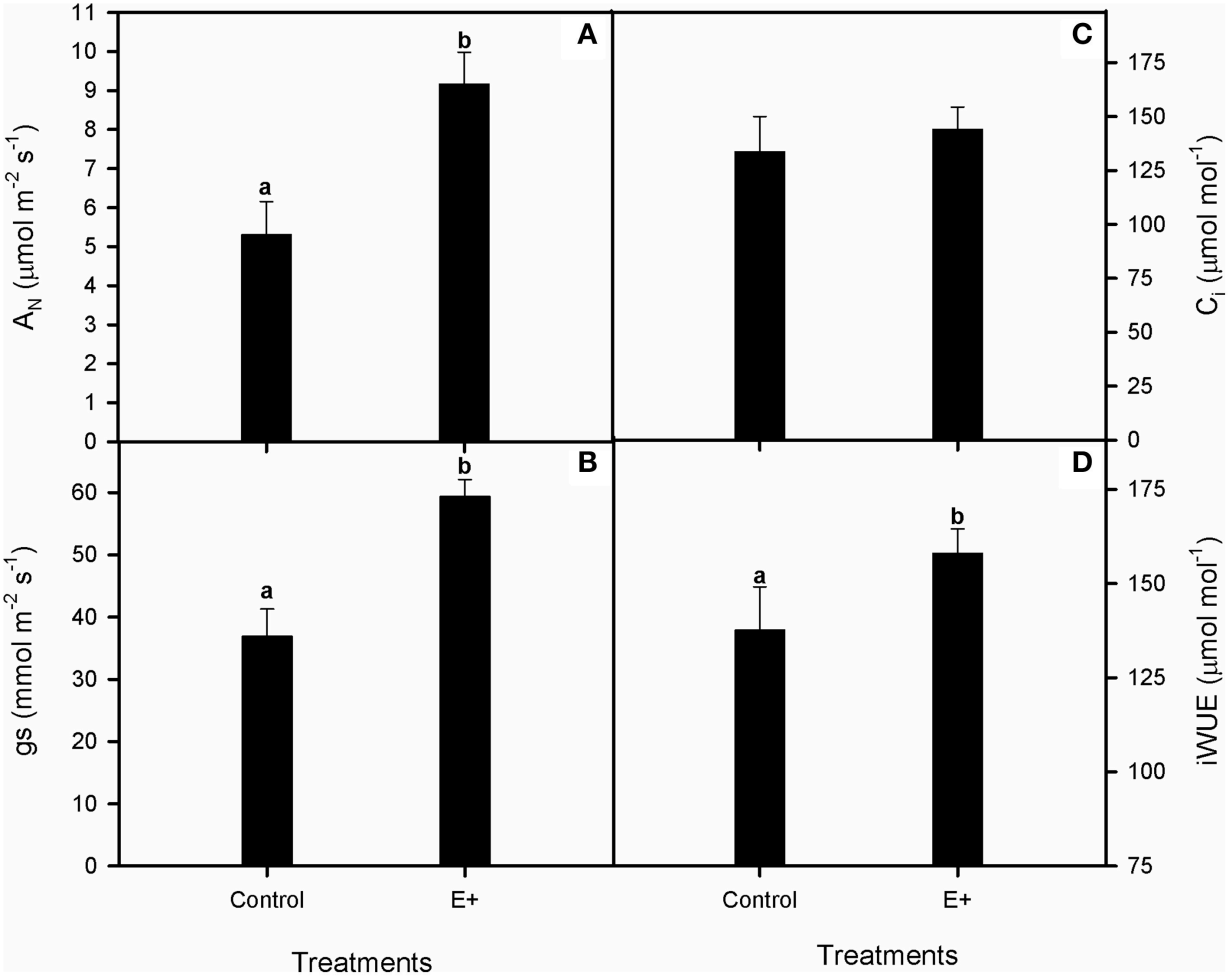
**Effect of inoculation with the endophytic bacterial consortium on net photosynthetic rate, A_N_ (A), stomatal conductance, gs (B), intercellular CO_2_ concentration, C_i_ (C) and intrinsic water-use efficiency, iWUE (D) in leaves of *Spartina maritima* plants grown in natural soil from Tinto marsh for 30 days**. Values are means ± s.e. (*n* = 10). Statistical differences between means are indicated by different letters (*P* < 0.05).

**Figure 6:**
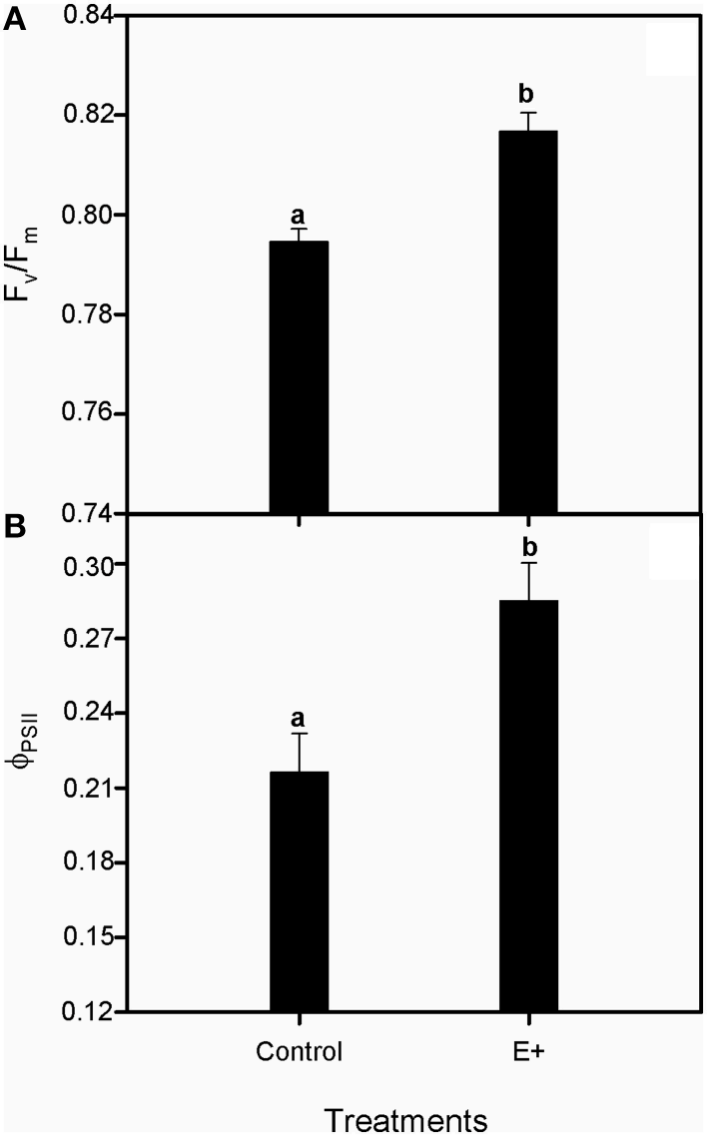
**Effect of inoculation with the endophytic bacterial consortium on maximum quantum efficiency of PSII photochemistry, F_v_/F_m_ (A) and quantum efficiency, Φ_PSII_ (B) in leaves of *Spartina maritima* plants grown in natural soil from Tinto marsh for 30 days**. Values are means ± s.e. (*n* = 10). Statistical differences between means are indicated by different letters (*P* < 0.05).

Plant inoculation with endophytes increased the relative growth rate (RGR) of *S. maritima* 25% after 30 days of treatment (*T*-test, *P* < 0.05; Figure 4A). This positive effect was restricted to belowground biomass increment (*T*-test, *P* < 0.05), whereas aboveground biomass did not vary respect to plants grown without bacterial inoculation (Figure 4B). Respect to gas exchange measurements, net photosynthetic rate (A_N_) values were greater in plants grown in soil inoculated with the endophytes (E+), with an increment of 42% after 30 days of treatment (*T*-test, *P* < 0.05; Figure 5A). Also stomatal conductance (gs) showed a similar trend to that of net photosynthetic rate (A_N_) (*T*-test, *P* < 0.05; Figure 5B). Whereas intercellular CO_2_ concentration (C_i_) values did not differ between treatments after 30 days of experiment, with values c. 135 μmol CO_2_ mol^−1^ air in both situations (Figure 5C). Furthermore, water use efficiency (iWUE) showed an increment of 15% in plants grown in inoculated soil (E+) after 30 days of treatment (*T*-test, *P* < 0.05; Figure 5D). Finally our fluorescence analysis showed that maximum quantum efficiency of PSII photochemistry (F_v_/F_m_) and quantum efficiency of PSII (Φ_PSII_) values at midday were greater in plants grown in soils inoculated with the endophytes (E+) after 30 days of treatment (*T*-test, *P* < 0.05; Figures 6A,B), whereas at dawn both parameters did not show significant differences between treatments with values c. 0.80 in all cases (data not shown).

### Ions concentrations in plant tissues after inoculation

**Figure 7:**
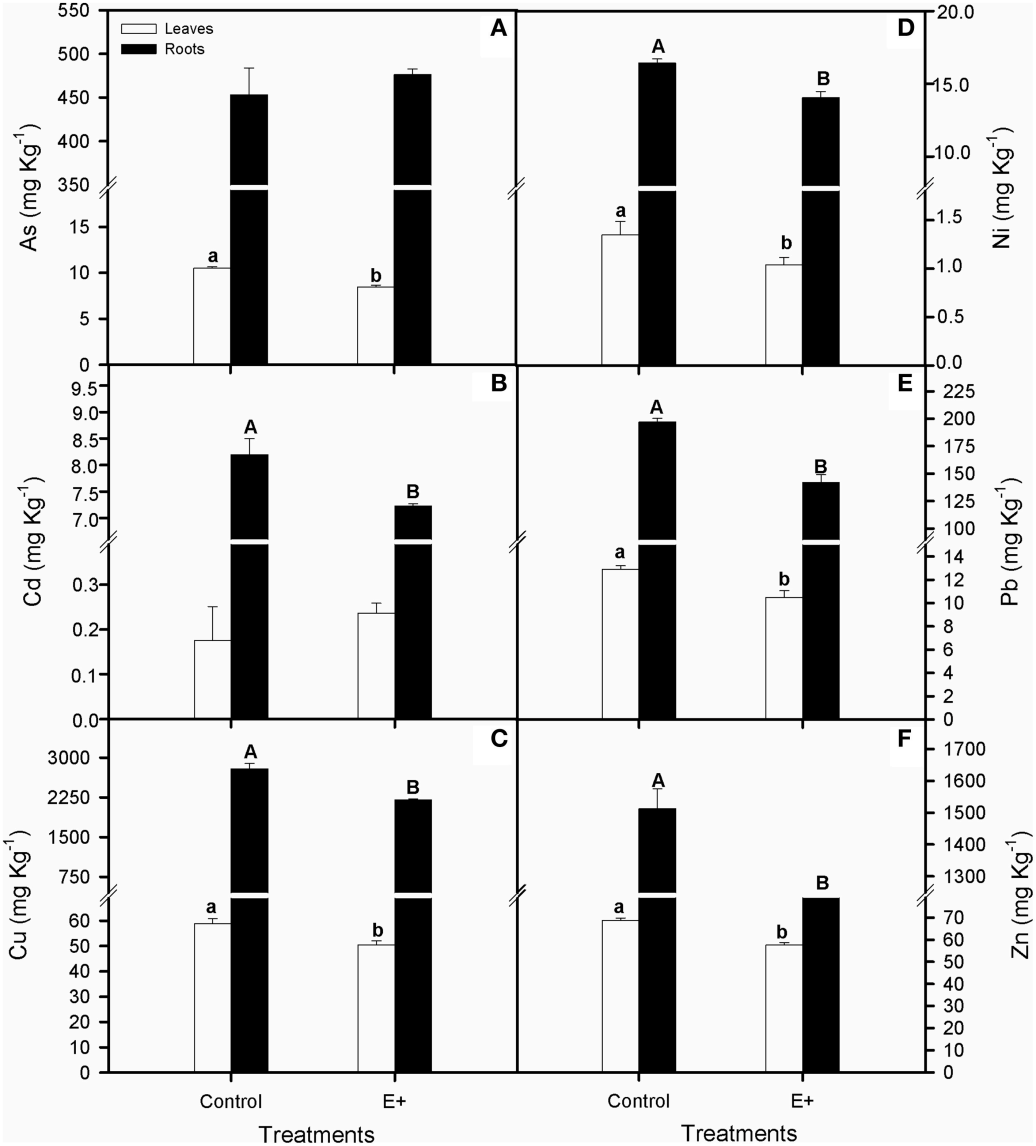
**Effect of inoculation with the endophytic bacterial consortium on total arsenic (A), cadmium (B), copper (C), nickel (D), lead (E), and zinc (F) for leaves and roots of *Spartina maritima* plants grown in natural soil from Tinto marsh for 30 days**. Values are means ± s.e. (*n* = 10). Different capital letters indicate statistical differences between inoculation treatments in roots and different lower case letters in leaves (*P* < 0.05).

At the end of the experiment, ion concentrations were greater in roots than in leaves of *S. maritima* in both treatments (*t*-test, *P* < 0.01; Figures 7A–F). Regarding the effect of soil inoculation on tissues ions concentrations, our results revealed that overall, concentration in roots and leaves decreased in plants grown in inoculated soil after 30 days of treatment (*T*-test, *P* < 0.05; Figures 7A–F). Thus, compared to the control, these decreases in roots and leaves ions concentrations (E+), were 22 and 14% for Cu, 15 and 24% for Ni, 28 and 19% for Pb and 19 and 17% for Zn, respectively. Decreases of 20% for As concentration in leaves and 12% for Cd in roots were also recorded. Nevertheless, considering the increments in biomass and the decrease in metal accumulation, no significant differences were observed in total metal balance in plants and soil between inoculated and non-inoculated plants (Supplementary Table 4).

### Assessment of endophytic colonization in *S. maritima* tissues

**Figure 8:**
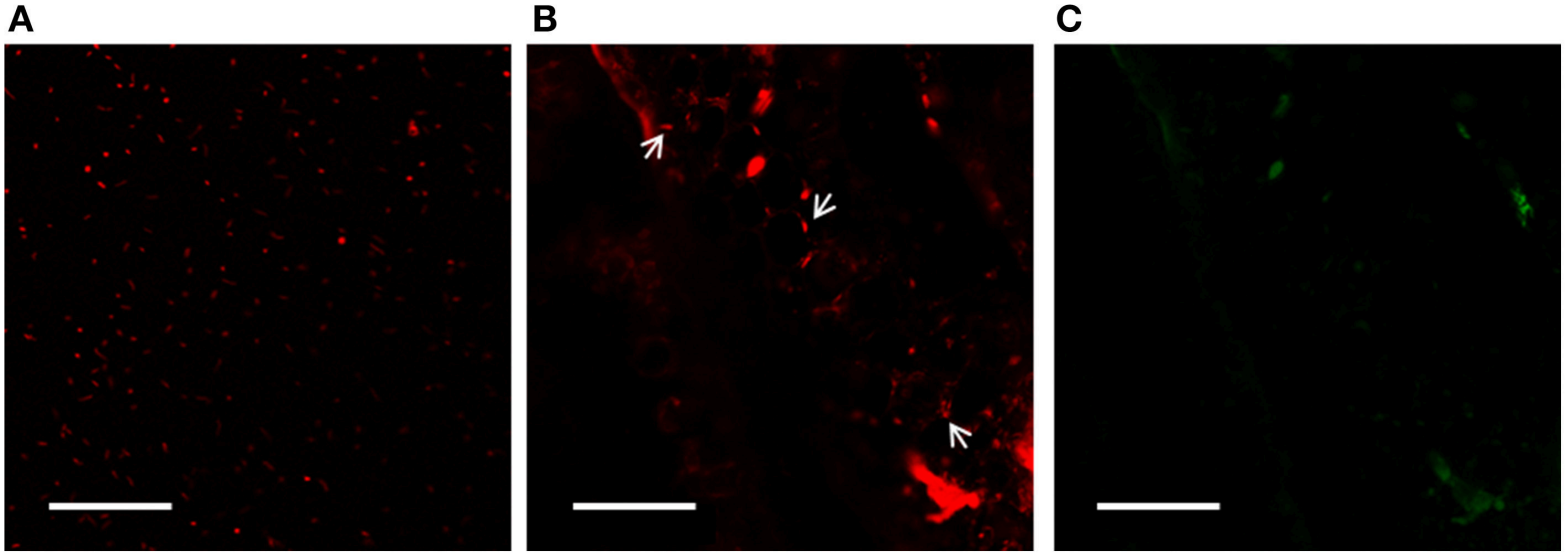
**CLSM analysis**. Images of mCherry-tagged *Salinicola peritrichatus* SMJ30 strains **(A)** and colonized *Spartina maritima* stems after 25 days of growth and inoculation with the endophytic consortium **(B, C)**. White arrows in **(B)** show bacteria colonizing the stem. Images were taken with different laser conditions to discriminate tissue fluorescence: **(B)** excitation 568–585 nm—long pass emission for red fluorescence; **(C)** excitation 488 nm—emission 522/35 nm for green fluorescence. Images were processed with ZEN Lite 2012 software. Scales bar represent 50 μm.

Using confocal laser scanning microscopy (CLSM), mCherry-tagged *Salinicola peritrichatus* SMJ30 cells were visualized from 0.5 mm slices of *S. maritima* stem (Figure 8). Roots and leaves fluoresced intensely, hence hindering the visualization of the bacteria. On the other hand, fluorescent colonies grew in TSA plates with rifampicin and tetracycline, while two different types of colonies grew in TSA plates amended with rifampicin and streptomycin. They were identified by morphological traits (macroscopic and microscopic observation) and biochemical traits (API® identification products (bioMérieux, France), obtaining the same results as the strains included in the original consortium. Hence, colonization of *S. maritima* tissues by the endophytic consortium was confirmed. In addition, CFU/gr of tissue and soil were estimated. For soil samples, no strains of SMJ30 or SMJ12 were detected in plates, while 3.5 × 10^2^ CFU of SMJ18 per gram were recorded. Roots were the most populated tissue by the endophytic consortium, as 3.4 × 10^2^ CFU of SMJ30, 2.1 × 10^3^ CFU of SMJ12 and 6.2 × 10^3^ CFU of SMJ18 per gram of tissue were estimated. Regarding the stem, 2.5 × 10^3^ and 2.2 × 10^2^ CFU/gr for SMJ30 and SMJ12, respectively, were registered whereas no colonies of SMJ18 appeared. Finally, leaves were the less re-colonized tissue, 30 and 90 CFU/gr for strains SMJ30 and SMJ12, respectively, were counted and, as the case of the stem, no colonies of SMJ18 were detected.

## Discussion

The estuarine sediments are important ecosystems that are largely influenced by plant activity (Almeida et al., 2006). In southwest coast of Spain, *S. maritima* is an endangered indigenous plant frequently used to restore degraded and contaminated salt marshes (Castillo and Figueroa, 2009). This species is included in European and National (Spanish) red lists which propose endangered species to be conserved (Cabezudo et al., 2005), since it is being displaced by the invasive *S. densiflora* (Castillo et al., 2008). Under these circumstances, intervention is needed. We propose the use of *S. maritima* and the cultivable bacteria associated to this plant as an ecological tool to regenerate contaminated marshes of southwest coast of Spain.

There is a extend literature on the use of PGPB as inoculants for improvement of plant growth in metal contaminated soils. One important conclusion that can be extracted from the analysis of previous results is that certain rhizospheric and endophytic bacteria have the ability to promote plant growth under stress conditions, but the effect of microbial inoculation on plant metal uptake cannot be predicted, since it depends on the specific plant-microbe partnerships and the characteristics of the soil and the contaminant (Sessitsch et al., 2013; Phieler et al., 2014; and references therein). For example, inoculation of plants with *Pseudomonas aeruginosa, P. fluorescens* and *Ralstonia metallidurans* strains enhanced Cr and Pb uptake by plants (Braud et al., 2009). Nevertheless, inoculation of *Phaseolus vulgaris* with a *Pseudomonas putida* strain reduced Cd and Pb accumulation in the plant (Tripathi et al., 2005).

The use of PGPR to promote the growth of *Spartina* plants in contaminated or degraded salt marshes have been recently proposed (Andrades-Moreno et al., 2014; Mateos-Naranjo et al., 2015; Mesa et al., 2015b). In one of these works, *S. maritima* wild plants were inoculated with indigenous PGP rhizobacteria (Mesa et al., 2015b). Rhizospheric bacteria increased the capacity of the plant to hyperaccumulate heavy metals by a variety of direct mechanisms, including enhanced heavy-metal mobilization and alleviation of heavy-metal toxicity to the plant, and indirect mechanisms comprising plant growth promotion and improved stress tolerance. Authors concluded that the rhizospheric consortium significantly enhanced the efficiency of metal rhizoaccumulation from natural soils by increasing both *S. maritima* belowground biomass and metal accumulation. Finally, the strategy was proposed as useful to enhance plant adaptation and metal rhizoaccumulation during marsh restoration programs.

Although scientists have mainly focused their research on plant-rhizobacteria interactions, endophytes may offer several competitive advantages over them, from their close and continued contact with plants. Bacterial endophytes are defined as those bacteria that colonize the inner parts of their host plants without causing disease symptoms (Hallmann et al., 1997; Schulz and Boyle, 2006). They have less competition from the surrounding microbes and the plant provides their nutrients. Furthermore, toxic pollutants taken up by the plant may be degraded in planta by endophytes reducing the toxic effects of contaminants in environmental soil on flora and fauna (Khan and Doty, 2011). On top of that, other beneficial effects attributed to endophytes include osmotic adjustment, stomatal regulation, modification of root morphology and enhanced uptake of minerals (Compant et al., 2005; Rajkumar et al., 2009). Finally, some metabolites are not only produced by a single organism, but might be produced by a plant associated with microorganisms (Brader et al., 2014). Hence, attention has focused in the last years on the role of endophytic bacteria in phytoremediation of contaminated soils (reviewed by Newman and Reynolds, 2005; Doty, 2008) and their use has been reported by several authors (reviewed in Rajkumar et al., 2009). The interactions between endophytes and hyperaccumulator plants have attracted the attention of several researchers, allowing the study of bacterial communities living on a naturally contaminated environment and their possible biotechnological applications for bioremediation (reviewed in Lodewyckx et al., 2002; Sessitsch et al., 2013; Visioli et al., 2014, 2015; Ma et al., 2015).

In this work, endophytic bacteria from the halophyte cordgrass *S. maritima* growing in polluted salt marshes in southwest Spain were studied. To our knowledge, this is the first work describing the endophytic populations of a halophyte growing in metal contaminated estuaries. On the whole, 25 strains were isolated. It was not surprising the extended bacterial resistance to copper, because is the most common heavy metal whether in soil (up to 3000 mg/kg) (Mesa et al., 2015a) or inside the plant (up to 545.47 mg/kg). Other prominent MTC values were observed for Co (30 mM), Ni (35 mM), Pb (28 mM) or As (100 mM) in the arsenite form, 4–100 times more toxic than arsenate. The amounts of arsenic found in plant tissue are generally proportional to its level in soil. It affects seed germination, and reduces root length and mass (Nie et al., 2002). Arsenic is one of the metal contaminants in soil that requires remediation (Consejería de Medio Ambiente, Junta de Andalucía, 1999), so further studies to describe the arsenite resistance mechanism in this isolate are being developed.

Endophytic enzymatic activities may aid in penetration and colonization of the host plant, as well as intervention in degradation of plant residues and plant nutrient acquisition (Wang and Dai, 2010). Among the isolates, 84% exhibited at least one hydrolytic enzyme activity out of five, thus demonstrating that endophytic bacteria can be an important source of a variety of enzymes. Concerning PGP properties, 76% of the strains had at least one of them. Endophytic PGPB may benefit plant growth increasing the accessibility or supply of major nutrients (Bashan, 1998). The production and modulation of auxins and ethylene play an essential role in plant development and stress tolerance (Brader et al., 2014). In addition, a well-studied form of biofertilization is nitrogen fixation, which is the conversion of atmospheric nitrogen to ammonia (Bloemberg and Lugtenberg, 2001). Moreover, some endophytic PGPB can increase phosphorus availability to the plant through phosphorus solubilization (Kpomblekou-A and Tabatabai, 2003). Finally, regarding biocontrol several mechanisms may be involved, including the production of siderophores or antibiotics (Gaiero et al., 2013).

Considering all the properties studied, the best performing endophytic strains were selected: *Micrococcus yunnanensis* SMJ12, *Vibrio sagamiensis* SMJ18, and *Salinicola peritrichatus* SMJ30. A mixture of naturally cohabitating endophytes may be a better alternative than applying an individual endophyte species, because different species may fulfill different ecological niches (Gaiero et al., 2013). It should also be emphasized that plant re-colonization would be apparently more feasible for endogenous bacteria than for exogenous ones. In this context, colonization of *S. maritima* tissues by the bacterial consortium was demonstrated. Strain SMJ30 was labeled with a fluorescent protein gene marker. This kind of genes has been widely used to visualize and track the colonization patterns of bacterial strains within inoculated host plants (Lagendijk et al., 2010). The presence of inoculated SMJ12 and SMJ18 strains was revealed using double antibiotic resistance selection, since it was not possible to tag these strains with marker genes. On the other hand, results suggested that different endophytic strains had preference for different plant tissues. For example, in this study strain SMJ18 was isolated from roots of *S. maritima* (Supplemetary Table 1) and after re-inoculation no presence of this strain in stems of leaves was observed. Strain SMJ12, isolated from *S. maritima* leaves (Supplemetary Table 1), was detected in all the tissues, probably due to the time spendt by the bacteria in its travel from the soil to the leaves. What is more, each strain is present in greater number in the tissue from which it was first isolated (SMJ18 in roots, SMJ30 in stems and SMJ12 in leaves). Even so, a greater number of CFU/gr was expected for leaves. Another remarkable point is the presence of the consortium in soil. Strain SMJ18, abundant in roots, was also growing in surrounding soil. However, SMJ12 and SMJ30, isolated from leaves and stem respectively (Supplementary Table 1), were not found in soil after 3 weeks of the last inoculation. These bacteria probably find better growing conditions inside the aerial part of the plant and leave the rhizospheric soil. This data may be considered preliminary, as a better study *in situ* in the salt marsh during a longer period of time is required to establish a well-founded assertion. Usually, greenhouse conditions limit several experimental procedures, as time or tidal flooding.

The inoculation with the selected endophytic bacterial consortium had a positive effect on the growth of *S. maritima*. Despite the fact that the RGR increased, this effect was restricted to belowground biomass. Root elongation has in fact been described as one of the major roles of PGPB (Glick, 2003). There is strong evidence that endophytic PGPB influences overall plant performance, but their detailed effects on photosynthesis, the basis of plant bio-chemical system, under metal stress is very scarce. Hence, in this study the effect of plant inoculation with the endophytic bacterial consortium on the photosynthetic apparatus of *S. maritima* was also analyzed. Obtained results suggested that the increase in growth can be attributed to the improvement in the photosynthetic carbon assimilation. Increased A_N_ values for the inoculation treatment were associated with an increment in gs. The joint increase in A_N_ and gs resulted in the augmentation of iWUE of inoculated plants. iWUE reflects the trade-off between CO_2_ acquisition for growth and water losses, and is therefore an important indicator of how plants manage water under stress conditions (Tardieu, 2012). Increased root-to-shoot ratio, through increased root growth in inoculated *S. maritima*, would contribute to increase the capacity for water absorption from the soil (Boyer, 1985) and, consequently, iWUE and metal tolerance. Also the functionality of PSII reflected a beneficial effect of inoculation with the endophytic bacterial consortium on the photosynthetic apparatus of *S. maritima*, as indicated by higher values of F_v_/F_m_ and Φ_PSII_ at midday, as well as increased chlorophyll pigments concentrations.

Moving on to metal accumulation, our results showed that the plant growth promotion caused by bacterial inoculation was also accompanied by an overall decrease in the concentration of metals in *S. maritima* tissues, denoting a lower rhizoaccumulation capacity than the one previously described (Cambrollé et al., 2008; Redondo-Gómez, 2012). These results suggest that the endophytic consortium appears to have a protective role against the presence of heavy metals in soil, lessening their uptake by *S. maritima* roots. Since plants were inoculated every week, this result could be due to a rizospheric effect of the bacteria before entering the plant. Metals could be complexed, precipitated and/or adsorbed onto bacterial surface in the rizosphere, thus reducing plant metal availability (reviewed in Sessitsch et al., 2013). Future research is needed to clarify mechanisms behind this effect. The decrement of metal concentration in tissues, as well as the presence of PGP properties in the bacterial consortium used in this experiment could explain the positive effect on *S. maritima* growth. Nevertheless, when total metal balance in plants and soil was estimated, no significant differences were observed between inoculated and non-inoculated plants, since the decrease in metal accumulation was accompanied by the increment in plant biomass. Furthermore, total As and Cd accumulated in inoculated plants was even higher than in non-inoculated plants after 1 month of plant growth (Supplementary Table 4).

In conclusion, we have designed an endophytic consortium that could be useful to promote *S. maritima* adaptation and growth in contaminated salt marshes. Inoculation with this consortium was not useful to increase plant metal accumulation, but could be a complementary strategy during marsh restoration programs, in situations where the increase of plant metal uptake is not desirable. For example, *S. maritima* plants could be first inoculated with the rhizospheric bacterial consortium previously described (Mesa et al., 2015b) and then, once they have loaded their roots with metals, inoculated with endophytic bacteria, facilitating plant growth without increasing metal content in roots in excess. In addition, inoculation with endophytes could also be an adequate strategy to promote *S. maritima* growth in non-contaminated salt-marshes, such as Piedras river estuary. Anyway, it is first necessary to test the effect of bacterial inoculation during longer periods, since conclusions until date have been extracted 30 days after inoculation. For this, a system to keep healthy *S. maritima* plants for more than 1 month in greenhouse conditions is being developed. In addition, an *in situ* experiment for marsh restoration in the south western coast of Spain, using autochthonous plants and the associated PGPB described in this work and those previously reported (Mesa et al., 2015b), is being designed.

## Author contributions

JM and EM performed most of the experimental work, data collection and analysis. JM, IR, and EM wrote the manuscript. All authors participated in the design of the study and took part in the evaluation of the results. All authors read and approved the final version of the manuscript to be published.

### Conflict of interest statement

The authors declare that the research was conducted in the absence of any commercial or financial relationships that could be construed as a potential conflict of interest.

## Footnotes

^1^USEPA. US Environmental Protection Agency. http://www.epa.gov
