# Supplementary material for: Endophytic Cultivable Bacteria of the Metal Bioaccumulator Spartina maritima Improve Plant Growth but Not Metal Uptake in Polluted Marshes Soils
Source: Front Microbiol. 2015 Dec 22;6:1450. doi: 10.3389/fmicb.2015.01450 (PMC4686625; doi:10.3389/fmicb.2015.01450)
Supplement: Supplementary file 1 [file Table1.DOCX]

| Strain |  | Morphology |  | Gram |  | Saltmarsh | | | | | | | | | | |
| --- | --- | --- | --- | --- | --- | --- | --- | --- | --- | --- | --- | --- | --- | --- | --- | --- |
|  |  |  |  |  |  | Tinto | | |  | Odiel | | |  | Piedras | | |
|  |  |  |  |  |  | Leave | Stem | Root |  | Leave | Stem | Root |  | Leave | Stem | Root |
| SMJ1 |  | Coccus |  | + |  | **·** |  |  |  | **·** |  |  |  | **·** |  |  |
| SMJ2 |  | Coccus |  | + |  | **·** | **·** |  |  |  | **·** |  |  |  | **·** |  |
| SMJ3 |  | Coccus |  | + |  | **·** |  |  |  |  |  |  |  |  |  |  |
| SMJ4 |  | Sarcinae |  | + |  | **·** |  |  |  | **·** |  |  |  | **·** |  |  |
| SMJ8 |  | Coccus |  | + |  |  |  |  |  | **·** |  |  |  |  |  |  |
| SMJ10 |  | Short bacillus |  | - |  |  |  |  |  | **·** |  |  |  |  |  |  |
| SMJ12 |  | Sarcinae |  | + |  | **·** |  |  |  | **·** |  |  |  |  |  |  |
| SMJ13 |  | Bacillus |  | + |  |  |  | **·** |  |  |  |  |  |  |  |  |
| SMJ14 |  | Long bacillus |  | - |  |  |  | **·** |  |  |  |  |  |  |  |  |
| *SMJ15* |  | Bacillus |  | + |  |  |  | **·** |  |  |  |  |  |  |  |  |
| SMJ16 |  | Bacillus |  | + |  |  |  | **·** |  |  |  |  |  |  |  |  |
| SMJ17 |  | Big bacillus |  | + |  |  |  | **·** |  |  |  |  |  |  |  |  |
| SMJ18 |  | Bacillus |  | - |  |  |  |  |  |  |  | **·** |  |  |  |  |
| SMJ19 |  | Short bacillus |  | - |  |  |  |  |  |  | **·** |  |  |  | **·** |  |
| *SMJ20* |  | Short bacillus |  | - |  |  |  |  |  |  |  | **·** |  |  |  |  |
| *SMJ21* |  | Bacillus |  | - |  |  | **·** | **·** |  |  | **·** | **·** |  |  |  |  |
| SMJ22 |  | Bacillus |  | - |  |  | **·** |  |  |  | **·** | **·** |  |  | **·** |  |
| SMJ24 |  | Bacillus |  | + |  |  |  |  |  |  |  |  |  |  |  | **·** |
| SMJ25 |  | Bacillus |  | + |  |  |  |  |  |  |  |  |  |  |  | **·** |
| SMJ26 |  | Bacillus |  | - |  |  |  |  |  |  |  |  |  |  |  | **·** |
| SMJ27 |  | Bacillus |  | - |  |  |  |  |  |  |  |  |  |  |  | **·** |
| *SMJ28* |  | Bacillus |  | + |  |  |  |  |  |  |  |  |  |  |  | **·** |
| SMJ30 |  | Bacillus |  | - |  |  | **·** |  |  |  | **·** |  |  |  | **·** |  |
| SMJ32 |  | Bacillus |  | - |  |  | **·** |  |  |  | **·** |  |  |  | **·** |  |
| SMJ33 |  | Coccus |  | + |  |  | **·** |  |  |  | **·** |  |  |  | **·** |  |

**Supplementary table 1.** Origin of the cultivable isolated endophytes.
