# Supplementary material for: Endophytic Cultivable Bacteria of the Metal Bioaccumulator Spartina maritima Improve Plant Growth but Not Metal Uptake in Polluted Marshes Soils
Source: Front Microbiol. 2015 Dec 22;6:1450. doi: 10.3389/fmicb.2015.01450 (PMC4686625; doi:10.3389/fmicb.2015.01450)
Supplement: Supplementary file 2 [file Table2.DOCX]

**Supplementary table 2.** Maximum tolerable concentration of heavy metals and NaCl for each isolate in ^a^ TSA and ^b^ TSA 0.2 M NaCl.

| Strain |  | Heavy metals (mM) | | | | | | | | | | | | | | | | | | | |  | NaCl  (M) |
| --- | --- | --- | --- | --- | --- | --- | --- | --- | --- | --- | --- | --- | --- | --- | --- | --- | --- | --- | --- | --- | --- | --- | --- |
|  |  | AsO^2-^ | |  | Cd ^2+^ | |  | Co^3+^ | |  | Cu^2+^ | |  | Ni^+^ | |  | Pb^2+^ | |  | Zn^2+^ | |  |  |
|  |  | a | b |  | a | b |  | a | b |  | a | b |  | a | b |  | a | b |  | a | b |  |  |
| SMJ1 |  | 1 | 0 |  | 0 | 0 |  | 2 | 3 |  | 6 | 7 |  | 6 | 17 |  | 25 | 25 |  | 3 | 3 |  | 3 |
| SMJ2 |  | 6 | 1 |  | 0 | 0 |  | 7 | 13 |  | 7 | 9 |  | 11 | 15 |  | 18 | 25 |  | 2 | 2 |  | 3 |
| SMJ3 |  | 25 | 17 |  | 0 | 0 |  | 7 | 13 |  | 5 | 6 |  | 6 | 12 |  | 25 | 28 |  | 1 | 1 |  | 3 |
| SMJ4 |  | 1 | 1 |  | 2 | 2 |  | 2 | 3 |  | 8 | 6 |  | 5 | 2 |  | 14 | 15 |  | 1 | 1 |  | 1 |
| SMJ8 |  | 1 | 1 |  | 0 | 0 |  | 2 | 7 |  | 2 | 7 |  | 3 | 10 |  | 20 | 10 |  | 2 | 2 |  | 3 |
| SMJ10 |  | 13 | 15 |  | 1 | 2 |  | 4 | 5 |  | 5 | 5 |  | 4 | 7 |  | 15 | 15 |  | 5 | 5 |  | 1 |
| SMJ12 |  | 100 | 17 |  | 2 | 2 |  | 1 | 1 |  | 9 | 5 |  | 2 | 3 |  | 15 | 13 |  | 2 | 2 |  | 1,5 |
| SMJ13 |  | 1 | 0 |  | 0 | 0 |  | 2 | 4 |  | 2 | 2 |  | 3 | 10 |  | 15 | 10 |  | 0 | 0 |  | 0,5 |
| SMJ14 |  | 2 | 0 |  | 0 | 0 |  | 1 | 0 |  | 2 | 0 |  | 1 | 0 |  | 5 | 5 |  | 0 | 0 |  | 0,5 |
| SMJ15 |  | 7 | 1 |  | 0 | 0 |  | 3 | 6 |  | 2 | 2 |  | 5 | 7 |  | 5 | 5 |  | 1 | 0 |  | 1 |
| SMJ16 |  | 1 | 0 |  | 0 | 0 |  | 2 | 3 |  | 2 | 2 |  | 3 | 5 |  | 10 | 10 |  | 0 | 0 |  | 1 |
| SMJ17 |  | 13 | 8 |  | 0 | 0 |  | 2 | 7 |  | 4 | 5 |  | 3 | 7 |  | 15 | 10 |  | 1 | 1 |  | 1 |
| SMJ18 |  | 4 | 1 |  | 1 | 6 |  | 2 | 30 |  | 5 | 6 |  | 5 | 35 |  | 10 | 12 |  | 5 | 6 |  | 1 |
| SMJ19 |  | 0 | 0 |  | 0 | 0 |  | 0 | 0 |  | 2 | 0 |  | 0 | 0 |  | 10 | 18 |  | 0 | 0 |  | 0,5 |
| SMJ20 |  | 0 | 1 |  | 0 | 0 |  | 0 | 6 |  | 0 | 6 |  | 0 | 10 |  | 20 | 21 |  | 0 | 2 |  | 1 |
| SMJ21 |  | 0 | 13 |  | 0 | 0 |  | 0 | 7 |  | 0 | 4 |  | 0 | 13 |  | 8 | 10 |  | 0 | 1 |  | 1 |
| SMJ22 |  | 0 | 0 |  | 0 | 0 |  | 0 | 0 |  | 2 | 0 |  | 0 | 0 |  | 5 | 5 |  | 0 | 0 |  | 0,5 |
| SMJ24 |  | 0 | 0 |  | 0 | 0 |  | 0 | 14 |  | 5 | 0 |  | 1 | 0 |  | 10 | 10 |  | 0 | 0 |  | 0,5 |
| SMJ25 |  | 0 | 0 |  | 0 | 0 |  | 0 | 14 |  | 1 | 4 |  | 0 | 8 |  | 10 | 10 |  | 0 | 6 |  | 1 |
| SMJ26 |  | 0 | 0 |  | 0 | 0 |  | 0 | 0 |  | 1 | 0 |  | 0 | 0 |  | 5 | 5 |  | 0 | 0 |  | 0,5 |
| SMJ27 |  | 0 | 0 |  | 0 | 0 |  | 0 | 4 |  | 1 | 1 |  | 0 | 9 |  | 9 | 10 |  | 0 | 0 |  | 1 |
| SMJ28 |  | 0 | 0 |  | 0 | 1 |  | 0 | 7 |  | 1 | 3 |  | 0 | 0 |  | 10 | 10 |  | 0 | 2 |  | 1 |
| SMJ30 |  | 5 | 4 |  | 0 | 1 |  | 3 | 13 |  | 4 | 9 |  | 1 | 13 |  | 5 | 25 |  | 7 | 8 |  | 3 |
| SMJ32 |  | 2 | 4 |  | 0 | 1 |  | 2 | 17 |  | 4 | 10 |  | 1 | 12 |  | 15 | 25 |  | 6 | 8 |  | 3 |
| SMJ33 |  | 1 | 1 |  | 0 | 0 |  | 3 | 12 |  | 8 | 8 |  | 2 | 13 |  | 22 | 25 |  | 1 | 1 |  | 2 |
