# Supplementary material for: Endophytic Cultivable Bacteria of the Metal Bioaccumulator Spartina maritima Improve Plant Growth but Not Metal Uptake in Polluted Marshes Soils
Source: Front Microbiol. 2015 Dec 22;6:1450. doi: 10.3389/fmicb.2015.01450 (PMC4686625; doi:10.3389/fmicb.2015.01450)
Supplement: Supplementary file 3 [file Table3.DOCX]

| **Strain** |  | **Enzymatic properties** | | | | | | | | |  | **PGP properties** | | | | | | | | |
| --- | --- | --- | --- | --- | --- | --- | --- | --- | --- | --- | --- | --- | --- | --- | --- | --- | --- | --- | --- | --- |
|  |  | **Amilase^b^** |  | **Cellulase^b^** |  | **Chitinase^b^** |  | **Protease^b^** |  | **Lipase^b^** |  | **ACC deaminase activity (μmoles α-ketobutyrate /h/mg)** |  | **Auxines production (mg/ml)** |  | **Nitrogen fixation^a^** |  | **Phosphates solubilisation^b^** |  | **Siderophores production^b^** |
| SMJ1 |  | - |  | - |  | - |  | - |  | - |  | - |  | - |  | + |  | + |  | + |
| SMJ2 |  | - |  | - |  | - |  | - |  | + |  | - |  | 0.82±0.14 |  | - |  | + |  | + |
| SMJ3 |  | - |  | - |  | - |  | - |  | - |  | - |  | - |  | - |  | - |  | - |
| SMJ4 |  | + |  | + |  | - |  | ++ |  | - |  | - |  | - |  | - |  | - |  | - |
| SMJ8 |  | - |  | - |  | - |  | - |  | + |  | - |  | - |  | + |  | + |  | - |
| SMJ10 |  | - |  | - |  | - |  | - |  | + |  | - |  | 1.31±0.21 |  | - |  | + |  | - |
| SMJ12 |  | - |  | + |  | - |  | ++ |  | - |  | - |  | 4.83±0.15 |  | - |  | + |  | + |
| SMJ13 |  | - |  | + |  | - |  | - |  | - |  | - |  | 2.61±0.13 |  | - |  | + |  | - |
| SMJ14 |  | + |  | + |  | - |  | - |  | + |  | - |  | - |  | - |  | - |  | + |
| SMJ15 |  | ++ |  | - |  | - |  | + |  | - |  | - |  | - |  | - |  | - |  | - |
| SMJ16 |  | - |  | - |  | - |  | + |  | - |  | - |  | - |  | - |  | - |  | - |
| SMJ17 |  | + |  | + |  | - |  | + |  | - |  | - |  | 3.29±0.09 |  | + |  | + |  | - |
| SMJ18 |  | ++ |  | ++ |  | + |  | ++ |  | + |  | - |  | 4.52±0.22 |  | - |  | - |  | + |
| SMJ19 |  | + |  | - |  | - |  | + |  | - |  | 3.21±0.05 |  | - |  | - |  | + |  | ++ |
| SMJ20 |  | + |  | + |  | - |  | + |  | + |  | ND |  | 5.18±0.21 |  | - |  | - |  | + |
| SMJ21 |  | ++ |  | + |  | - |  | ++ |  | - |  | - |  | - |  | - |  | + |  | + |
| SMJ22 |  | - |  | - |  | - |  | - |  | - |  | - |  | - |  | - |  | - |  | - |
| SMJ24 |  | - |  | + |  | + |  | + |  | + |  | - |  | 1.13±0.34 |  | - |  | - |  | + |
| SMJ25 |  | + |  | + |  | + |  | + |  | ++ |  | - |  | - |  | - |  | - |  | + |
| SMJ26 |  | - |  | - |  | - |  | - |  | - |  | - |  | - |  | - |  | - |  | - |
| SMJ27 |  | + |  | - |  | - |  | - |  | - |  | - |  | 2.55±0.08 |  | - |  | - |  | - |
| SMJ28 |  | + |  | - |  | - |  | + |  | - |  | 3.42±0.09 |  | 1.93±0.08 |  | + |  | + |  | ++ |
| SMJ30 |  | + |  | - |  | - |  | - |  | + |  | - |  | - |  | - |  | + |  | ++ |
| SMJ32 |  | + |  | - |  | - |  | - |  | + |  | - |  | - |  | - |  | ++ |  | + |
| SMJ33 |  | - |  | - |  | - |  | - |  | + |  | - |  | 2.12±0.17 |  | + |  | + |  | - |

**Supplementary table 3.** Enzymatic and plant growth promoting properties of the bacterial endophytic *S. maritima* isolates.

^a^ The results are expressed by presence (+) or absence (-) of visible growth

^b^ The results are expressed by presence (+) or absence (-) of halo

+: halos < 10 mm ++: halos ≥ 10 mm

ND: not determined
