# Supplementary material for: Endophytic Cultivable Bacteria of the Metal Bioaccumulator Spartina maritima Improve Plant Growth but Not Metal Uptake in Polluted Marshes Soils
Source: Front Microbiol. 2015 Dec 22;6:1450. doi: 10.3389/fmicb.2015.01450 (PMC4686625; doi:10.3389/fmicb.2015.01450)
Supplement: Supplementary file 4 [file Table4.DOCX]

**Supplementary Table 4.** Metal balance in plant and soil (%) before and after the inoculation experiment. E+, inoculations repeated once a week during experimental period.

| Metal/loid | Initial | | Final | | | |
| --- | --- | --- | --- | --- | --- | --- |
|  |  |  | Control | | E+ | |
|  | Plant | Soil | Plant | Soil | Plant | Soil |
| As | 0,63 | 99,37 | 1,05 | 98,95 | 1,34 | 98,66 |
| Cd | 0,78 | 99,22 | 1,97 | 98,03 | 2,11 | 97,89 |
| Cu | 0,56 | 99,44 | 1,43 | 98,57 | 1,37 | 98,63 |
| Ni | 0,26 | 99,74 | 0,50 | 99,50 | 0,51 | 99,49 |
| Pb | 0,2 | 99,8 | 0,37 | 99,63 | 0,32 | 99,68 |
| Zn | 0,5 | 99,5 | 0,77 | 99,23 | 0,76 | 99,24 |
